# Supplementary material for: Excess cost of care associated with sepsis in cancer patients: Results from a population-based case-control matched cohort
Source: PLoS One. 2021 Aug 11;16(8):e0255107. doi: 10.1371/journal.pone.0255107 (PMC8357157; doi:10.1371/journal.pone.0255107)
Supplement: S1 Appendix — (DOCX) [file pone.0255107.s001.docx]

**S1 Appendix: Data source**

Health care administrative databases containing information on all Ontario residents used for this study were held at and accessed through the Institute for Clinical Evaluative Sciences (ICES).

Table A1: List of datasets and costing methodology

| **Dataset** | **Information / Type of health care service** | **Resource use information for costing** | **Cost estimation methodology** |
| --- | --- | --- | --- |
| **Ontario Cancer Registry** | Used to identify cancer cohort, type of cancer diagnosed | - | - |
| **Registered Person Database** | Mortality (date of death) and baseline demographics (e.g. age, sex, socioeconomic status) | - | - |
| **Canadian Institute for Health Information-Discharge abstract** | Inpatient hospitalisation & same day surgery | Resource intensity weight (RIW) | RIW * Unit cost per weighted case [1] |
| **Canadian Institute for Health Information-National ambulatory care reporting system** | Ambulatory care – emergency department, cancer clinic and dialysis clinic | Resource intensity weight (RIW) | RIW * Unit cost per weighted case [1] |
| **Ontario Health Insurance Plan claims** | All physician services including primary care consultations, specialist consultations, allied health services, diagnostic tests and laboratory services | Costs reported in dataset | Costs as per provided in dataset. However, for physicians that were shadow-billed (reported cost in dataset = $0), costs were imputed using the mean cost of the fee-for-service records of the same year and fee code [1, 2] |
| **Continuing Care reporting system** | Other institution-based care; e.g. rehabilitation, complex continuing care and long-term care | Utilisation intensity weight and length of stay (LOS) | Utilisation intensity weight * LOS * per diem cost [1] |
| **Ontario Drug Benefit program** | Outpatient prescriptions | Costs reported in dataset | Costs as per provided in dataset |
| **New Drug Funding Program** | Chemotherapy supplied | Costs reported in dataset | Costs as per provided in dataset |
| **Activity level reporting system** | Radiation therapy | National Hospital Productivity Improvement Program (NHPIP) codes | Intensity of resource use (minutes) from NHPIP codes * cost per min [1, 3] |

The costing approaches used in this analysis followed the comprehensive guidance provided by Wodchis et al. [1] for costing healthcare services using administrative datasets specific to Ontario. Costs for inpatient hospitalisations, emergency department and ambulatory care visits and long-term care were estimated by multiplying resource intensity weight (RIW) by cost per weighted case or day. Each RIW represents an average measure of resource use by individuals based on their case mix with a particular condition relative to the average resource used by other patients. In Ontario, these weights have been developed by Canadian Institute for Health Information (CIHI). Units costs specific to each type of healthcare service were derived based on the Ontario Cost Distribution Methodology (developed by the Health Data Branch of the Ontario Ministry of Health and Long-Term Care). Unit costs across the years of interest specific to each type of healthcare service were sourced from Wodchis et al. and ICES for our cost calculations. Costs for medications, chemotherapy and physician services were available directly in the data. Radiation costs were based on the intensity of resource use captured by National Hospital Productivity Improvement Program (NHPIP) codes and unit cost obtained from Earle et al.[3]. All costs were then adjusted to 2018 dollars using the healthcare component of the Statistics Canada Consumer Price Index.

**References**

1. Wodchis WP, Bushmeneva K, Nikitovic M, et al. Guidelines on person-level costing using administrative databases in Ontario. 2013.

2. de Oliveira C, Bremner KE, Liu N, et al. Costs for childhood and adolescent cancer, 90 days prediagnosis and 1 year postdiagnosis: A population-based study in Ontario, Canada. Value in Health. 2017; 20: 345-56.

3. Earle C, Coyle D, Smith A, et al. The cost of radiotherapy at an Ontario regional cancer centre: a re-evaluation. Critical reviews in oncology/hematology. 1999; 32: 87-93.
