# Supplementary material for: Excess cost of care associated with sepsis in cancer patients: Results from a population-based case-control matched cohort
Source: PLoS One. 2021 Aug 11;16(8):e0255107. doi: 10.1371/journal.pone.0255107 (PMC8357157; doi:10.1371/journal.pone.0255107)
Supplement: S2 Appendix — (DOCX) [file pone.0255107.s002.docx]

**S2 Appendix: Diagnostic codes used for identification of sepsis**

Sepsis cases were identified using codes from the 10^th^ (ICD-10-CA) Revisions. Cases were classified within two mutually exclusive groups, “explicit” and “implicit.” Explicit sepsis cases were those with an ICD code explicitly referencing sepsis listed as an admission diagnosis. Implicit sepsis cases were those with both an infection code and organ dysfunction code listed as admission diagnoses. Any cases captured through either explicit or implicit codes were considered to be a sepsis case.

Table A2: ICD-10-CA codes used in the identification of sepsis sourced from the Global Burden of Disease Study^[[1]](#footnote-1)^

| **Explicit** | **Implicit** | |
| --- | --- | --- |
|  | **Infection codes** | **Organ dysfunction codes** |
| A02.1-A02.9, A20.7-A20.9, A21.7-A21.9, A22.7-A22.9, A24.1-A24.9, A26.7-A26.9, A28.2-A28.9, A32.7-A32.9, A39.0, A39.4-A41.9, A42.7- A42.9, A50-A50.9, A54.86, B00.7-B00.9, B37.7-B37.9, N98.0, O03.0, O03.3, O03.5, O03.8, O04.5, O04.8, O07.3, O08.0, O08.83, O23-O23.9, O41.1-O41.9, O75.3, O85- O86.8, O88.3-O88.3, O91- O91.23, O98, O98.2-O98.9, P00.2, P22-P23.9, P29.1, P29.8, P35-P37, P37.1-P39.9, R65.2-R65.2, R68.1 | A01-A02.0, A03-A09.9, A19-A20.3, A21-A21.3, A22-A22.2, A23-A24.0, A25-A26.0, A27-A28.1, A31-A32.12, A36-A39, A39.1-A39.3, A42-A42.2, A43-A46.0, A48-A49.9, A59-A59.9, A65-A65.0, A69-A69.1, A74, A74.8- A75.9, A77-A81.9, A83-A96.9, A98-B00.59, B01-B10.89, B25-B27.99,  B29.4, B33-B34.9, B37-B37.6, B38-B50.9, B54-B55, B55.1-B55.9, B58-  B60.8, B64, B67-B67.99, B91, B95-B99.9, G00-G08.0, G14-G14.6, H05.01-H05.039, H60.2-H60.23, H70.0-H70.009, I00, I02, I02.9, I26.01-I26.09, I26.90-I26.99, I33-I33.9, I38-I39.9, I40.0-I40.9, I76, I96-I96.9, I98.1, J01-J06.9, J09-J22.9, J36-J36.0, J39.0-J39.1, J85-J86.9, K35-K37.9, K57-K57.93, K61-K61.4, K63.0-K63.1, K65-K65.9, K67.8, K75.0-K75.1, K75.3, K76.3, K77.0, K81.0, K81.2, K83.0, K95.01, K95.81, L02-L08.9, M00-M02.9, M86-M86.9, M89.6-M89.69, N10-N10.9, N15.1-N15.9, N30-N30.91, N39.0, N41.0, N41.2-N41.3, N45-N45.9, N70-N77.8, R78.81, T80.2-T80.29, T81.4, T82.6-T82.7, T83.5, T83.6, T84.5-T84.7, T85.7, T88.0, U04 | D65-D65.9, D69.5-D69.59, E87.2-E87.99, G93.4-G93.49, I46-I46.9, I95.1-I95.9, J80-J80.9, J95.2-J95.3, J96- J96.92, K72-K72.91, N00- N01.9, N17-N17.9, R09.02, R09.2, R40.0-R40.4, R41.82, R55-R55.0, R57-R57.9 |

Within the Ontario Health Insurance Plan (OHIP) dataset, diagnosis was captured using a separate set of diagnostic codes and sepsis was identified using the diagnosis code “038 – Septicaemia”.

1. Rudd KE, Johnson SC, Agesa KM, et al. Global, regional, and national sepsis incidence and mortality, 1990–2017: analysis for the Global Burden of Disease Study. The Lancet. 2020; 395: 200-11. [↑](#footnote-ref-1)
