# Supplementary material for: Excess cost of care associated with sepsis in cancer patients: Results from a population-based case-control matched cohort
Source: PLoS One. 2021 Aug 11;16(8):e0255107. doi: 10.1371/journal.pone.0255107 (PMC8357157; doi:10.1371/journal.pone.0255107)
Supplement: S3 Appendix — (DOCX) [file pone.0255107.s003.docx]

**S3 Appendix: Distribution of sepsis episodes from time of cancer diagnosis**

Figure A1: Proportion of sepsis episodes among cases at each monthly interval from time of cancer diagnosis. Patients with multiple sepsis episodes were included in multiple intervals but only once per time interval.
