# Supplementary material for: Excess cost of care associated with sepsis in cancer patients: Results from a population-based case-control matched cohort
Source: PLoS One. 2021 Aug 11;16(8):e0255107. doi: 10.1371/journal.pone.0255107 (PMC8357157; doi:10.1371/journal.pone.0255107)

**S4 Appendix: Kaplan-Meier survival curves comparing sepsis cases to no sepsis controls**

Figure A2 shows Kaplan-Meier survival curves comparing sepsis cases to no sepsis controls. Mortality was high among sepsis cases, particularly in the first year of cancer diagnosis. The 5-year overall survival rate for haematology patients with sepsis was 29.1%(95% CI,28.2-30.0) and 66.5%(95% CI,65.6-67.4) for those without sepsis. In the solid tumour cancer group, the 5-year overall survival for patients with and without sepsis was 28.4%(95% CI,28.0-28.8) and 54.2%(95% CI,53.8-54.6), respectively. The difference in five-year overall survival between cancer patients with sepsis and without sepsis was statistically significant (log rank test p<0.001) across both cancer types.

Figure A2: Kaplan-Meier survival curves
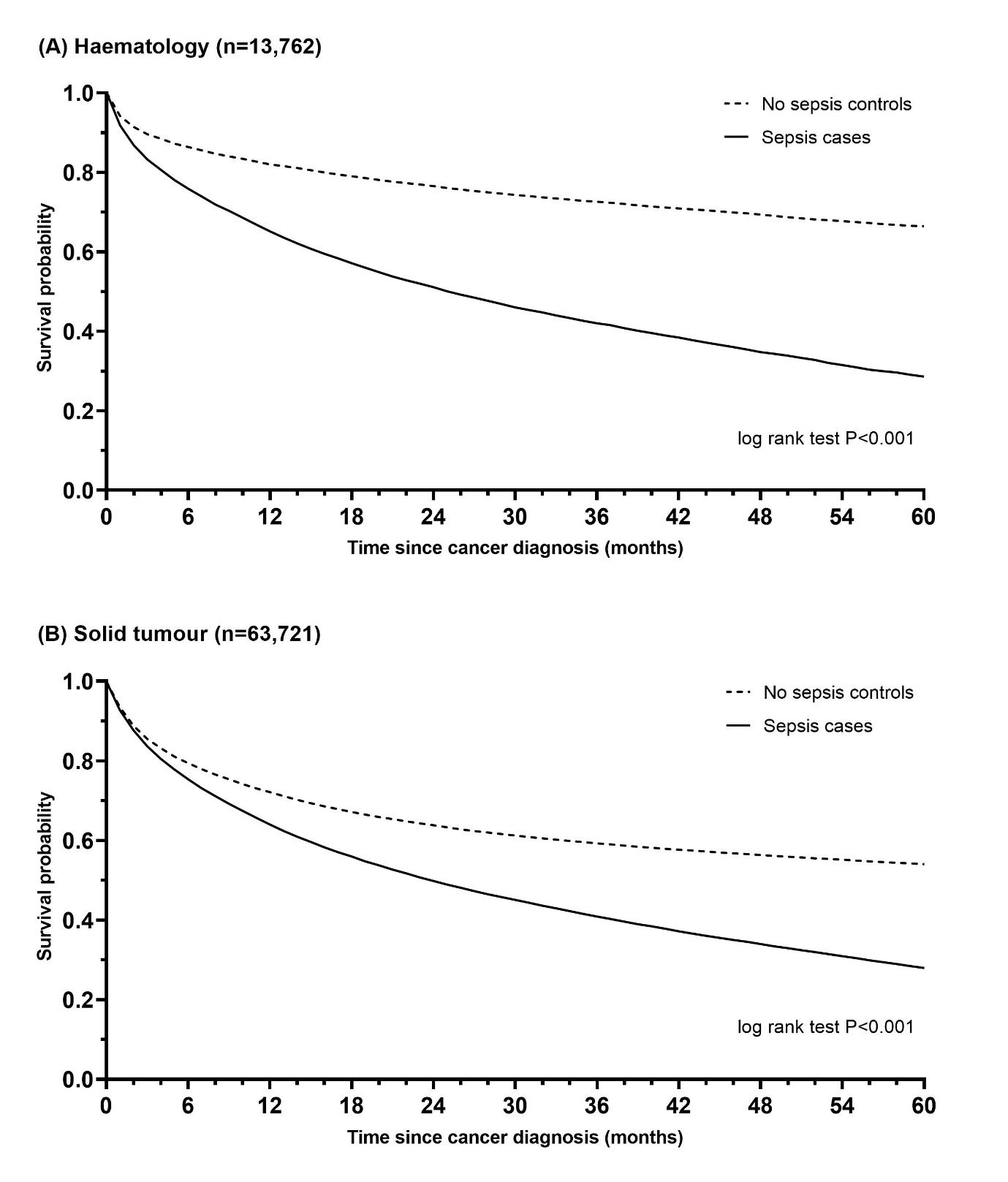

Supplement: S4 Appendix — (DOCX) [file pone.0255107.s004.docx]
