# Supplementary material for: Excess cost of care associated with sepsis in cancer patients: Results from a population-based case-control matched cohort
Source: PLoS One. 2021 Aug 11;16(8):e0255107. doi: 10.1371/journal.pone.0255107 (PMC8357157; doi:10.1371/journal.pone.0255107)
Supplement: S5 Appendix — (DOCX) [file pone.0255107.s005.docx]

**S5 Appendix: Descriptive statistics of sepsis cases vs. matched controls by malignancy types**

Table A3: Characteristics of sepsis cases vs. matched controls for haematology patients

| **Characteristic** | **Haematology** | | | |  |
| --- | --- | --- | --- | --- | --- |
|  | **Sepsis cases** | | **Matched controls (no sepsis)** | |  |
|  | **Number**  **(N = 13,762)** | **Percent** | **Number**  **(N = 13,762)** | **Percent** | **P-value** |
| Age | | | | | P=0.996 |
| 18-34 | 496 | 3.6 | 497 | 3.61 |  |
| 35-44 | 554 | 4.03 | 575 | 4.18 |  |
| 45-54 | 1,343 | 9.76 | 1,334 | 9.69 |  |
| 55-64 | 2,541 | 18.46 | 2,563 | 18.62 |  |
| 65-74 | 3,486 | 25.33 | 3,458 | 25.13 |  |
| 75-84 | 3,521 | 25.58 | 3,514 | 25.53 |  |
| 85+ | 1,821 | 13.23 | 1,821 | 13.23 |  |
| Female | 6,115 | 44.43 | 6,115 | 44.43 | P=1.000 |
| Urban/rural residence | | | | | P=1.000 |
| Urban | 12,236 | 88.91 | 12,236 | 88.91 |  |
| Rural | 1,526 | 11.09 | 1,526 | 11.09 |  |
| Income quintile | | | | | P<0.001 |
| Low | 2,878 | 20.96 | 2,636 | 19.21 |  |
| Medium-low | 2,955 | 21.52 | 2,815 | 20.52 |  |
| Medium | 2,679 | 19.51 | 2,666 | 19.43 |  |
| Medium-high | 2,628 | 19.14 | 2,678 | 19.52 |  |
| High | 2,590 | 18.86 | 2,926 | 21.32 |  |
| Type of cancer | | | | | P=1.000 |
| Leukaemia | 8,174 | 59.40 | 8,174 | 59.40 |  |
| Lymphoma | 3,367 | 24.47 | 3,367 | 24.47 |  |
| Myeloma | 2,221 | 16.14 | 2,221 | 16.14 |  |
| Year of cancer diagnosis | | | | | P=1.000 |
| 2010 | 1,767 | 12.84 | 1,767 | 12.84 |  |
| 2011 | 1,698 | 12.34 | 1,698 | 12.34 |  |
| 2012 | 1,725 | 12.53 | 1,725 | 12.53 |  |
| 2013 | 1,772 | 12.88 | 1,772 | 12.88 |  |
| 2014 | 1,799 | 13.07 | 1,799 | 13.07 |  |
| 2015 | 1,855 | 13.48 | 1,855 | 13.48 |  |
| 2016 | 1,694 | 12.31 | 1,694 | 12.31 |  |
| 2017 | 1,452 | 10.55 | 1,452 | 10.55 |  |

Table A4: Characteristics of sepsis cases vs. matched controls for solid tumour patients

| **Characteristic** | **Solid tumour** | | | |  |
| --- | --- | --- | --- | --- | --- |
|  | **Sepsis cases** | | **Matched controls (no sepsis)** | |  |
|  | **Number**  **(N = 63,721)** | **Percent** | **Number**  **(N = 63,721)** | **Percent** | **P-value** |
| Age | | | | | P=0.728 |
| 18-34 | 964 | 1.51 | 973 | 1.53 |  |
| 35-44 | 1,962 | 3.08 | 1,953 | 3.06 |  |
| 45-54 | 6,141 | 9.64 | 6,338 | 9.95 |  |
| 55-64 | 13,655 | 21.43 | 13,627 | 21.39 |  |
| 65-74 | 18,639 | 29.25 | 18,551 | 29.11 |  |
| 75-84 | 15,821 | 24.83 | 15,740 | 24.7 |  |
| 85+ | 6,539 | 10.26 | 6,539 | 10.26 |  |
| Female | 29,765 | 46.71 | 29,765 | 46.71 | P=1.000 |
| Urban/rural residence | | | | | P=1.000 |
| Urban | 56,034 | 88.29 | 56,034 | 88.29 |  |
| Rural | 7,473 | 11.73 | 7,473 | 11.73 |  |
| Income quintile | | | | | P<0.001 |
| Low | 14,509 | 22.83 | 12,780 | 20.1 |  |
| Medium-low | 13,788 | 21.69 | 13,164 | 20.71 |  |
| Medium | 12,531 | 19.71 | 12,701 | 19.98 |  |
| Medium-high | 11,675 | 18.37 | 12,492 | 19.65 |  |
| High | 11,060 | 17.4 | 12,432 | 19.56 |  |
| Type of cancer | | | | | P=1.000 |
| Lung | 11,601 | 18.21 | 11,601 | 18.21 |  |
| Colorectal | 10,415 | 16.34 | 10,415 | 16.34 |  |
| Breast ^b^ | 6,271 | 9.84 | 6,271 | 9.84 |  |
| Prostate | 5,565 | 8.73 | 5,565 | 8.73 |  |
| Bladder | 2,929 | 4.6 | 2,929 | 4.6 |  |
| Pancreatic | 2,627 | 4.12 | 2,627 | 4.12 |  |
| Stomach | 2,224 | 3.49 | 2,224 | 3.49 |  |
| Head and neck | 2,220 | 3.48 | 2,220 | 3.48 |  |
| Kidney | 1,960 | 3.08 | 1,960 | 3.08 |  |
| Liver | 1,916 | 3.01 | 1,916 | 3.01 |  |
| Melanoma | 1,812 | 2.84 | 1,812 | 2.84 |  |
| Uterus | 1,705 | 2.68 | 1,705 | 2.68 |  |
| Ovary | 1,395 | 2.19 | 1,395 | 2.19 |  |
| Brain | 1,066 | 1.67 | 1,066 | 1.67 |  |
| Oesophagus | 1,044 | 1.64 | 1,044 | 1.64 |  |
| Thyroid | 666 | 1.05 | 666 | 1.05 |  |
| Cervical | 506 | 0.79 | 506 | 0.79 |  |
| Testis | 181 | 0.28 | 181 | 0.28 |  |
| Others | 7,618 | 11.96 | 7,618 | 11.96 |  |
| Year of cancer diagnosis | | | | | P=1.000 |
| 2010 | 7,881 | 12.37 | 7,881 | 12.37 |  |
| 2011 | 8,441 | 13.25 | 8,441 | 13.25 |  |
| 2012 | 8,670 | 13.61 | 8,670 | 13.61 |  |
| 2013 | 8,925 | 14.01 | 8,925 | 14.01 |  |
| 2014 | 8,524 | 13.38 | 8,524 | 13.38 |  |
| 2015 | 8,145 | 12.78 | 8,145 | 12.78 |  |
| 2016 | 7,571 | 11.88 | 7,571 | 11.88 |  |
| 2017 | 5,564 | 8.73 | 5,564 | 8.73 |  |
