# Supplementary material for: Excess cost of care associated with sepsis in cancer patients: Results from a population-based case-control matched cohort
Source: PLoS One. 2021 Aug 11;16(8):e0255107. doi: 10.1371/journal.pone.0255107 (PMC8357157; doi:10.1371/journal.pone.0255107)
Supplement: S6 Appendix — (DOCX) [file pone.0255107.s006.docx]

**S6 Appendix: Descriptive statistics of sepsis cases vs. unmatched cases by malignancy types**

Table A5: Characteristics of sepsis cases vs. unmatched cases for haematology patients

| **Characteristic** | **Haematology** | | | |  |
| --- | --- | --- | --- | --- | --- |
|  | **Sepsis cases** | | **Unmatched cases** | |  |
|  | **Number**  **(N = 13,762)** | **Percent** | **Number**  **(N = 2,763)** | **Percent** | **P-value** |
| Age | | | | | P<0.001 |
| 18-34 | 496 | 3.6 | 116 | 4.2 |  |
| 35-44 | 554 | 4.03 | 70 | 2.53 |  |
| 45-54 | 1,343 | 9.76 | 173 | 6.26 |  |
| 55-64 | 2,541 | 18.46 | 433 | 15.67 |  |
| 65-74 | 3,486 | 25.33 | 671 | 24.29 |  |
| 75-84 | 3,521 | 25.58 | 810 | 29.32 |  |
| 85+ | 1,821 | 13.23 | 490 | 17.73 |  |
| Female | 6,115 | 44.43 | 1,139 | 41.22 | P=0.002 |
| Urban/rural residence | | | | | P<0.001 |
| Urban | 12,236 | 88.91 | 2,522 | 91.22 |  |
| Rural | 1,526 | 11.09 | 241 | 8.78 |  |
| Income quintile | | | | | P=0.309 |
| Low | 2,878 | 20.96 | 567 | 20.7 |  |
| Medium-low | 2,955 | 21.52 | 553 | 20.19 |  |
| Medium | 2,679 | 19.51 | 551 | 20.12 |  |
| Medium-high | 2,628 | 19.14 | 514 | 18.77 |  |
| High | 2,590 | 18.86 | 554 | 20.23 |  |
| Type of cancer | | | | | P<0.001 |
| Leukaemia | 8,174 | 59.40 | 1,575 | 57 |  |
| Lymphoma | 3,367 | 24.47 | 356 | 12.88 |  |
| Myeloma | 2,221 | 16.14 | 832 | 30.11 |  |
| Year of cancer diagnosis | | | | | P<0.001 |
| 2010 | 1,767 | 12.84 | 418 | 15.13 |  |
| 2011 | 1,698 | 12.34 | 416 | 15.06 |  |
| 2012 | 1,725 | 12.53 | 478 | 17.3 |  |
| 2013 | 1,772 | 12.88 | 482 | 17.44 |  |
| 2014 | 1,799 | 13.07 | 385 | 13.93 |  |
| 2015 | 1,855 | 13.48 | 329 | 11.91 |  |
| 2016 | 1,694 | 12.31 | 180 | 6.51 |  |
| 2017 | 1,452 | 10.55 | 75 | 2.71 |  |
| Outcome at end of study period | | | | |  |
| Died | 8,831 | 64.17 | 1,910 | 69.13 | P<0.001 |

Table A6: Characteristics of sepsis cases vs. unmatched cases for solid tumour patients

| **Characteristic** | **Solid tumour** | | | |  |
| --- | --- | --- | --- | --- | --- |
|  | **Sepsis cases** | | **Unmatched cases** | |  |
|  | **Number**  **(N = 63,721)** | **Percent** | **Number**  **(N = 2,782)** | **Percent** | **P-value** |
| Age | | | | | P<0.001 |
| 18-34 | 964 | 1.51 | 88 | 3.16 |  |
| 35-44 | 1,962 | 3.08 | 74 | 2.66 |  |
| 45-54 | 6,141 | 9.64 | 148 | 5.32 |  |
| 55-64 | 13,655 | 21.43 | 359 | 12.9 |  |
| 65-74 | 18,639 | 29.25 | 721 | 25.92 |  |
| 75-84 | 15,821 | 24.83 | 907 | 32.6 |  |
| 85+ | 6,539 | 10.26 | 485 | 17.43 |  |
| Female | 29,765 | 46.71 | 1,044 | 37.53 | P<0.001 |
| Urban/rural residence | | | | | P=0.240 |
| Urban | 56,034 | 88.29 | 2,408 | 87.53 |  |
| Rural | 7,473 | 11.73 | 343 | 12.47 |  |
| Income quintile | | | | | P=0.058 |
| Low | 14,509 | 22.83 | 589 | 21.47 |  |
| Medium-low | 13,788 | 21.69 | 566 | 20.63 |  |
| Medium | 12,531 | 19.71 | 593 | 21.62 |  |
| Medium-high | 11,675 | 18.37 | 498 | 18.16 |  |
| High | 11,060 | 17.4 | 497 | 18.12 |  |
| Type of cancer | | | | | P<0.001 |
| Lung | 11,601 | 18.21 | 357 | 12.83 |  |
| Colorectal | 10,415 | 16.34 | 486 | 17.47 |  |
| Breast ^a^ | 6,271 | 9.84 | 32 | 1.15 |  |
| Prostate | 5,565 | 8.73 | 110 | 3.95 |  |
| Bladder | 2,929 | 4.6 | 358 | 12.87 |  |
| Pancreatic | 2,627 | 4.12 | 228 | 8.2 |  |
| Stomach | 2,224 | 3.49 | 173 | 6.22 |  |
| Head and neck | 2,220 | 3.48 | 50 | 1.8 |  |
| Kidney | 1,960 | 3.08 | 66 | 2.37 |  |
| Liver | 1,916 | 3.01 | 257 | 9.24 |  |
| Melanoma | 1,812 | 2.84 | 33 | 1.19 |  |
| Uterus | 1,705 | 2.68 | 18 | 0.65 |  |
| Ovary | 1,395 | 2.19 | 38 | 1.37 |  |
| Brain | 1,066 | 1.67 | 40 | 1.44 |  |
| Oesophagus | 1,044 | 1.64 | 93 | 3.34 |  |
| Thyroid | 666 | 1.05 | 13 | 0.47 |  |
| Cervical | 506 | 0.79 | 20 | 0.72 |  |
| Testis | 181 | 0.28 | 18 | 0.65 |  |
| Others | 7,618 | 11.96 | 392 | 14.09 |  |
| Year of cancer diagnosis | | | | | P<0.001 |
| 2010 | 7,881 | 12.37 | 326 | 11.72 |  |
| 2011 | 8,441 | 13.25 | 356 | 12.8 |  |
| 2012 | 8,670 | 13.61 | 459 | 16.5 |  |
| 2013 | 8,925 | 14.01 | 520 | 18.69 |  |
| 2014 | 8,524 | 13.38 | 421 | 15.13 |  |
| 2015 | 8,145 | 12.78 | 366 | 13.16 |  |
| 2016 | 7,571 | 11.88 | 239 | 8.59 |  |
| 2017 | 5,564 | 8.73 | 95 | 3.41 |  |
| Outcome at end of study period | | | | | |
| Died | 42,357 | 66.47 | 2,145 | 77.1 | P<0.001 |

^a^ Breast cancer among females
