# Supplementary material for: Excess cost of care associated with sepsis in cancer patients: Results from a population-based case-control matched cohort
Source: PLoS One. 2021 Aug 11;16(8):e0255107. doi: 10.1371/journal.pone.0255107 (PMC8357157; doi:10.1371/journal.pone.0255107)
Supplement: S7 Appendix — (DOCX) [file pone.0255107.s007.docx]

**S7 Appendix: Breakdown of excess cost of care due to sepsis**

Figure A3: Mean monthly excess cost of care due to sepsis by malignancy type. Black solid line represents monthly mean excess costs and the red shared area represents terminal care cost (last 6 months).
