# Supplementary material for: Excess cost of care associated with sepsis in cancer patients: Results from a population-based case-control matched cohort
Source: PLoS One. 2021 Aug 11;16(8):e0255107. doi: 10.1371/journal.pone.0255107 (PMC8357157; doi:10.1371/journal.pone.0255107)
Supplement: S8 Appendix — (DOCX) [file pone.0255107.s008.docx]

**S8 Appendix: Sub-group analyses results (by sex and age categories)**

Table A7: Cumulative cost of care ($CAD 2018) between sepsis cases and matched controls among females

| **Time since cancer diagnosis (months)** | **Haematology (Female)** | | | | | | | | |
| --- | --- | --- | --- | --- | --- | --- | --- | --- | --- |
|  | **Sepsis cases** | | | **Matched controls** | | | **Excess cost** | | |
|  | **Mean** | **95% CI** | | **Mean** | **95% CI** | | **Mean** | **95% CI** | |
|  |  | **LL** | **UL** |  | **LL** | **UL** |  | **LL** | **UL** |
| 1 | 18,881 | 18,586 | 19,177 | 7,116 | 6,942 | 7,290 | 11,765 | 11,416 | 12,114 |
| 3 | 34,262 | 33,883 | 34,641 | 13,810 | 13,597 | 14,022 | 20,452 | 20,016 | 20,888 |
| 6 | 53,478 | 52,986 | 53,970 | 23,080 | 22,816 | 23,344 | 30,398 | 29,835 | 30,961 |
| 12 | 78,626 | 78,027 | 79,224 | 34,165 | 33,846 | 34,484 | 44,461 | 43,774 | 45,148 |
| 24 | 107,489 | 106,807 | 108,172 | 48,503 | 48,136 | 48,871 | 58,986 | 58,201 | 59,771 |
| 60 | 157,059 | 156,184 | 157,933 | 82,892 | 82,338 | 83,446 | 74,166 | 73,146 | 75,187 |

| **Time since cancer diagnosis (months)** | **Solid tumour (Female)** | | | | | | | | |
| --- | --- | --- | --- | --- | --- | --- | --- | --- | --- |
|  | **Sepsis cases** | | | **Matched controls** | | | **Excess cost** | | |
|  | **Mean** | **95% CI** | | **Mean** | **95% CI** | | **Mean** | **95% CI** | |
|  |  | **LL** | **UL** |  | **LL** | **UL** |  | **LL** | **UL** |
| 1 | 17,069 | 16,770 | 17,369 | 9,501 | 9,351 | 9,652 | 7,568 | 7,233 | 7,904 |
| 3 | 34,362 | 33,973 | 34,751 | 21,145 | 20,925 | 21,366 | 13,217 | 12,774 | 13,660 |
| 6 | 51,286 | 50,835 | 51,736 | 32,629 | 32,349 | 32,910 | 18,656 | 18,134 | 19,178 |
| 12 | 72,642 | 72,129 | 73,154 | 44,588 | 44,246 | 44,931 | 28,053 | 27,446 | 28,660 |
| 24 | 93,571 | 92,982 | 94,160 | 54,918 | 54,531 | 55,305 | 38,653 | 37,957 | 39,349 |
| 60 | 131,394 | 130,657 | 132,131 | 73,543 | 73,064 | 74,022 | 57,851 | 56,989 | 58,713 |

Table A8: Cumulative cost of care ($CAD 2018) between sepsis cases and matched controls among males

| **Time since cancer diagnosis (months)** | **Haematology (Male)** | | | | | | | | |
| --- | --- | --- | --- | --- | --- | --- | --- | --- | --- |
|  | **Sepsis cases** | | | **Matched controls (no sepsis)** | | | **Excess cost** | | |
|  | **Mean** | **95% CI** | | **Mean** | **95% CI** | | **Mean** | **95% CI** | |
|  |  | **LL** | **UL** |  | **LL** | **UL** |  | **LL** | **UL** |
| 1 | 19,463 | 19,141 | 19,786 | 6,939 | 6,786 | 7,092 | 12,524 | 12,170 | 12,879 |
| 3 | 35,922 | 35,528 | 36,315 | 14,625 | 14,425 | 14,825 | 21,297 | 20,854 | 21,740 |
| 6 | 56,239 | 55,740 | 56,738 | 24,483 | 24,242 | 24,725 | 31,756 | 31,204 | 32,309 |
| 12 | 82,709 | 82,112 | 83,306 | 36,127 | 35,825 | 36,428 | 46,583 | 45,918 | 47,248 |
| 24 | 112,218 | 111,537 | 112,899 | 51,067 | 50,708 | 51,426 | 61,151 | 60,391 | 61,912 |
| 60 | 162,082 | 161,234 | 162,930 | 85,493 | 84,951 | 86,035 | 76,589 | 75,588 | 77,589 |

| **Time since cancer diagnosis (months)** | **Solid tumour (Male)** | | | | | | | | |
| --- | --- | --- | --- | --- | --- | --- | --- | --- | --- |
|  | **Sepsis cases** | | | **Matched controls (no sepsis)** | | | **Excess cost** | | |
|  | **Mean** | **95% CI** | | **Mean** | **95% CI** | | **Mean** | **95% CI** | |
|  |  | **LL** | **UL** |  | **LL** | **UL** |  | **LL** | **UL** |
| 1 | 18,015 | 17,682 | 18,348 | 10,058 | 9,894 | 10,221 | 7,957 | 7,587 | 8,328 |
| 3 | 37,309 | 36,858 | 37,760 | 22,697 | 22,457 | 22,937 | 14,611 | 14,089 | 15,134 |
| 6 | 54,874 | 54,338 | 55,409 | 33,354 | 33,065 | 33,642 | 21,520 | 20,894 | 22,145 |
| 12 | 73,562 | 72,962 | 74,163 | 42,991 | 42,670 | 43,313 | 30,571 | 29,880 | 31,262 |
| 24 | 95,732 | 95,054 | 96,411 | 53,457 | 53,099 | 53,816 | 42,275 | 41,493 | 43,057 |
| 60 | 135,851 | 135,006 | 136,697 | 71,915 | 71,472 | 72,358 | 63,936 | 62,970 | 64,903 |

Table A9: Cumulative cost of care ($CAD 2018) between haematology sepsis cases and matched controls by age categories

| **Time since cancer diagnosis (months)** | **Haematology (Age 18-34)** | | | | | | | | |
| --- | --- | --- | --- | --- | --- | --- | --- | --- | --- |
|  | **Sepsis cases** | | | **Matched controls (no sepsis)** | | | **Excess cost** | | |
|  | **Mean** | **95% CI** | | **Mean** | **95% CI** | | **Mean** | **95% CI** | |
|  |  | **LL** | **UL** |  | **LL** | **UL** |  | **LL** | **UL** |
| 1 | 26,543 | 26,038 | 27,047 | 7,331 | 7,139 | 7,523 | 19,212 | 18,681 | 19,742 |
| 3 | 46,633 | 46,047 | 47,219 | 13,633 | 13,413 | 13,853 | 33,000 | 32,392 | 33,609 |
| 6 | 71,611 | 70,892 | 72,330 | 23,201 | 22,912 | 23,490 | 48,410 | 47,643 | 49,177 |
| 12 | 100,093 | 99,281 | 100,905 | 31,899 | 31,559 | 32,238 | 68,194 | 67,321 | 69,068 |
| 24 | 122,815 | 121,894 | 123,735 | 37,218 | 36,855 | 37,582 | 85,596 | 84,614 | 86,579 |
| 60 | 146,932 | 145,816 | 148,047 | 43,974 | 43,596 | 44,351 | 102,958 | 101,781 | 104,134 |
|  | **Haematology (Age 35-44)** | | | | | | | | |
| 1 | 27,644 | 27,109 | 28,178 | 5,721 | 5,560 | 5,883 | 21,922 | 21,359 | 22,486 |
| 3 | 46,253 | 45,669 | 46,837 | 12,598 | 12,386 | 12,810 | 33,655 | 33,025 | 34,286 |
| 6 | 70,313 | 69,614 | 71,012 | 21,150 | 20,895 | 21,406 | 49,163 | 48,412 | 49,914 |
| 12 | 99,545 | 98,707 | 100,383 | 28,936 | 28,624 | 29,249 | 70,609 | 69,709 | 71,509 |
| 24 | 123,203 | 22,254 | 124,152 | 34,915 | 34,581 | 35,250 | 88,287 | 87,280 | 89,294 |
| 60 | 150,125 | 149,082 | 151,169 | 44,165 | 43,797 | 44,534 | 105,960 | 104,851 | 107,069 |
|  | **Haematology (Age 45-54)** | | | | | | | | |
| 1 | 24,943 | 24,448 | 25,438 | 5,474 | 5,324 | 5,625 | 19,469 | 18,947 | 19,990 |
| 3 | 43,392 | 42,818 | 43,966 | 12,373 | 12,180 | 12,567 | 31,019 | 30,407 | 31,630 |
| 6 | 68,321 | 67,585 | 69,058 | 21,204 | 20,962 | 21,447 | 47,117 | 46,343 | 47,891 |
| 12 | 97,304 | 96,394 | 98,213 | 29,578 | 29,275 | 29,881 | 67,726 | 66,765 | 68,686 |
| 24 | 123,026 | 122,013 | 124,039 | 36,665 | 36,338 | 36,992 | 86,361 | 85,295 | 87,426 |
| 60 | 156,246 | 155,112 | 157,380 | 52,025 | 51,626 | 52,424 | 104,221 | 103,010 | 105,431 |
|  | **Haematology (Age 55-64)** | | | | | | | | |
| 1 | 21,522 | 21,120 | 21,923 | 5,913 | 5,732 | 6,094 | 15,608 | 15,167 | 16,049 |
| 3 | 40,664 | 40,145 | 41,183 | 12,830 | 12,623 | 13,036 | 27,834 | 27,279 | 28,389 |
| 6 | 64,255 | 63,640 | 64,870 | 22,101 | 21,856 | 22,346 | 42,154 | 41,494 | 42,815 |
| 12 | 93,558 | 92,798 | 94,319 | 31,427 | 31,127 | 31,727 | 62,132 | 61,317 | 62,946 |
| 24 | 121,625 | 120,727 | 122,523 | 41,986 | 41,627 | 42,346 | 79,639 | 78,662 | 80,615 |
| 60 | 166,346 | 165,334 | 167,359 | 65,631 | 65,159 | 66,102 | 100,715 | 99,577 | 101,854 |
|  | **Haematology (Age 65-74)** | | | | | | | | |
| 1 | 18,387 | 18,058 | 18,716 | 6,200 | 6,044 | 6,357 | 12,187 | 11,828 | 12,546 |
| 3 | 35,181 | 34,762 | 35,600 | 13,996 | 13,796 | 14,196 | 21,185 | 20,713 | 21,657 |
| 6 | 54,685 | 54,198 | 55,173 | 24,562 | 24,309 | 24,814 | 30,124 | 29,559 | 30,688 |
| 12 | 81,656 | 81,058 | 82,253 | 37,067 | 36,759 | 37,375 | 44,589 | 43,895 | 45,283 |
| 24 | 14,394 | 113,702 | 115,086 | 53,933 | 53,568 | 54,298 | 60,461 | 59,661 | 61,262 |
| 60 | 172,429 | 171,562 | 173,296 | 97,012 | 96,435 | 97,589 | 75,417 | 74,361 | 76,474 |
|  | **Haematology (Age 75-84)** | | | | | | | | |
| 1 | 16,153 | 15,879 | 16,428 | 7,369 | 7,218 | 7,520 | 8,785 | 8,468 | 9,101 |
| 3 | 29,494 | 29,172 | 29,816 | 14,885 | 14,684 | 15,086 | 14,609 | 14,232 | 14,986 |
| 6 | 45,127 | 44,754 | 45,499 | 24,245 | 24,007 | 24,482 | 20,882 | 20,443 | 21,322 |
| 12 | 67,166 | 66,712 | 67,620 | 37,073 | 36,792 | 37,353 | 30,093 | 29,559 | 30,628 |
| 24 | 95,795 | 95,253 | 96,337 | 56,118 | 55,741 | 56,494 | 39,678 | 39,008 | 40,348 |
| 60 | 159,212 | 158,354 | 160,070 | 106,427 | 105,746 | 107,108 | 52,785 | 51,691 | 53,879 |
|  | **Haematology (Age 85+)** | | | | | | | | |
| 1 | 15,516 | 15,322 | 15,709 | 10,686 | 10,525 | 10,847 | 4,830 | 4,582 | 5,077 |
| 3 | 25,162 | 24,924 | 25,399 | 17,609 | 17,412 | 17,807 | 7,552 | 7,243 | 7,862 |
| 6 | 37,738 | 37,437 | 38,039 | 27,731 | 27,469 | 27,994 | 10,007 | 9,605 | 10,408 |
| 12 | 57,390 | 56,963 | 57,816 | 43,365 | 43,014 | 43,716 | 11,782 | 10,084 | 13,479 |
| 24 | 90,308 | 89,751 | 90,865 | 71,445 | 70,953 | 71,937 | 14,024 | 13,475 | 14,574 |
| 60 | 160,594 | 159,410 | 161,779 | 148,812 | 147,618 | 150,006 | 18,863 | 18,121 | 19,606 |

Table A10: Cumulative cost of care ($CAD 2018) between solid tumour sepsis cases and matched controls by age categories

| **Time since cancer diagnosis (months)** | **Solid tumour (Age 18-34)** | | | | | | | | |
| --- | --- | --- | --- | --- | --- | --- | --- | --- | --- |
|  | **Sepsis cases** | | | **Matched controls (no sepsis)** | | | **Excess cost** | | |
|  | **Mean** | **95% CI** | | **Mean** | **95% CI** | | **Mean** | **95% CI** | |
|  |  | **LL** | **UL** |  | **LL** | **UL** |  | **LL** | **UL** |
| 1 | 20,342 | 19,636 | 21,049 | 7,540 | 7,382 | 7,698 | 12,803 | 12,069 | 13,536 |
| 3 | 39,657 | 38,844 | 40,470 | 17,266 | 17,034 | 17,499 | 22,391 | 21,539 | 23,242 |
| 6 | 58,618 | 57,663 | 59,573 | 26,554 | 26,236 | 26,871 | 32,064 | 31,068 | 33,061 |
| 12 | 81,086 | 80,094 | 82,079 | 37,268 | 36,887 | 37,648 | 43,819 | 42,757 | 44,880 |
| 24 | 100,188 | 99,141 | 101,234 | 43,622 | 43,219 | 44,025 | 56,566 | 55,441 | 57,691 |
| 60 | 126,715 | 125,552 | 127,878 | 54,177 | 53,719 | 54,634 | 72,539 | 71,293 | 73,785 |
|  | **Solid tumour (Age 35-44)** | | | | | | | | |
| 1 | 16,672 | 16,360 | 16,985 | 7,999 | 7,845 | 8,153 | 8,673 | 8,324 | 9,023 |
| 3 | 35,677 | 35,243 | 36,111 | 19,591 | 19,338 | 19,843 | 16,086 | 15,596 | 16,577 |
| 6 | 53,634 | 53,126 | 54,142 | 30,911 | 30,606 | 31,216 | 22,723 | 22,143 | 23,302 |
| 12 | 81,789 | 81,181 | 82,396 | 45,132 | 44,754 | 45,509 | 36,657 | 35,972 | 37,342 |
| 24 | 102,269 | 101,606 | 102,931 | 52,682 | 52,277 | 53,087 | 49,587 | 48,834 | 50,339 |
| 60 | 132,553 | 131,787 | 133,319 | 62,949 | 62,514 | 63,384 | 69,604 | 68,745 | 70,464 |
|  | **Solid tumour (Age 45-54)** | | | | | | | | |
| 1 | 16,761 | 16,439 | 17,083 | 9,113 | 8,917 | 9,310 | 7,647 | 7,274 | 8,020 |
| 3 | 36,964 | 36,526 | 37,403 | 22,674 | 22,382 | 22,967 | 14,290 | 13,761 | 14,819 |
| 6 | 55,091 | 54,588 | 55,593 | 34,073 | 33,739 | 34,408 | 21,017 | 20,396 | 21,638 |
| 12 | 79,098 | 78,526 | 79,669 | 46,224 | 45,846 | 46,602 | 32,874 | 32,171 | 33,577 |
| 24 | 99,798 | 99,166 | 100,429 | 53,772 | 53,365 | 54,180 | 46,025 | 45,264 | 6,786 |
| 60 | 131,390 | 130,643 | 132,138 | 64,980 | 64,519 | 65,441 | 66,410 | 65,511 | 67,309 |
|  | **Solid tumour (Age 55-64)** | | | | | | | | |
| 1 | 17,140 | 16,818 | 17,462 | 9,429 | 9,257 | 9,602 | 7,711 | 7,348 | 8,073 |
| 3 | 36,982 | 36,551 | 37,413 | 22,797 | 22,539 | 23,055 | 14,185 | 13,681 | 14,689 |
| 6 | 55,963 | 55,433 | 56,492 | 34,147 | 33,834 | 34,459 | 21,816 | 21,196 | 22,436 |
| 12 | 77,051 | 76,444 | 77,658 | 44,896 | 44,539 | 45,253 | 32,155 | 31,442 | 32,868 |
| 24 | 98,557 | 97,876 | 99,238 | 53,899 | 53,516 | 54,283 | 44,657 | 43,857 | 45,457 |
| 60 | 133,984 | 133,159 | 134,809 | 68,481 | 68,035 | 68,927 | 65,503 | 64,554 | 66,451 |
|  | **Solid tumour (Age 65-74)** | | | | | | | | |
| 1 | 17,675 | 17,330 | 18,020 | 9,690 | 9,520 | 9,860 | 7,985 | 7,597 | 8,372 |
| 3 | 36,550 | 36,113 | 36,988 | 22,581 | 22,321 | 22,840 | 13,970 | 13,451 | 14,488 |
| 6 | 54,886 | 54,362 | 55,409 | 34,473 | 34,158 | 34,788 | 20,413 | 19,795 | 21,031 |
| 12 | 74,595 | 73,979 | 75,211 | 44,569 | 44,214 | 44,925 | 30,026 | 29,310 | 30,742 |
| 24 | 95,856 | 95,161 | 96,550 | 54,596 | 54,199 | 54,992 | 41,260 | 40,452 | 42,069 |
| 60 | 135,891 | 135,026 | 136,755 | 72,383 | 71,923 | 72,843 | 63,507 | 62,531 | 64,484 |
|  | **Solid tumour (Age 75-84)** | | | | | | | | |
| 1 | 17,897 | 17,568 | 18,226 | 10,369 | 10,179 | 10,559 | 7,528 | 7,139 | 7,918 |
| 3 | 34,847 | 34,375 | 35,318 | 21,635 | 21,384 | 21,886 | 13,212 | 12,679 | 13,745 |
| 6 | 50,144 | 49,628 | 50,660 | 31,796 | 31,506 | 32,086 | 18,348 | 17,752 | 18,944 |
| 12 | 66,996 | 66,416 | 67,577 | 41,436 | 41,106 | 41,765 | 25,561 | 24,885 | 26,237 |
| 24 | 88,887 | 88,236 | 89,537 | 52,768 | 52,391 | 53,146 | 36,118 | 35,354 | 36,882 |
| 60 | 134,325 | 133,440 | 135,210 | 77,195 | 76,690 | 77,700 | 57,130 | 56,112 | 58,148 |
|  | **Solid tumour (Age 85+)** | | | | | | | | |
| 1 | 18,136 | 17,832 | 18,440 | 11,373 | 11,232 | 11,514 | 6,763 | 6,428 | 7,099 |
| 3 | 30,487 | 30,102 | 30,873 | 20,360 | 20,161 | 20,558 | 10,127 | 9,689 | 10,565 |
| 6 | 42,248 | 41,805 | 42,691 | 29,075 | 28,824 | 29,326 | 13,173 | 12,665 | 13,680 |
| 12 | 57,902 | 57,407 | 58,397 | 40,844 | 40,522 | 41,166 | 27,781 | 26,655 | 28,906 |
| 24 | 81,831 | 81,237 | 82,424 | 59,043 | 58,609 | 59,476 | 17,058 | 16,471 | 17,646 |
| 60 | 133,159 | 132,322 | 133,996 | 105,378 | 104,616 | 106,140 | 22,788 | 22,051 | 23,526 |
