# Supplementary material for: Excess cost of care associated with sepsis in cancer patients: Results from a population-based case-control matched cohort
Source: PLoS One. 2021 Aug 11;16(8):e0255107. doi: 10.1371/journal.pone.0255107 (PMC8357157; doi:10.1371/journal.pone.0255107)
Supplement: S9 Appendix — (DOCX) [file pone.0255107.s009.docx]

**S9 Appendix: Sensitivity analyses**

The following sensitivity analyses were conducted, and their results presented in the tables below.

1. Including income quintiles and additional socioeconomic scores (dependency, deprivation, ethnic concentration) as matching variables to identify controls (at the expense of identifying suitable controls)
2. Excluding the 1-month pre-diagnosis period from our sepsis case definition (total number of sepsis cases = 75,785)
3. Alternate case definition of sepsis (Sepsis-2) [1] using Jolley’s et al. ICD-10-coded case definition [2]
4. Duration attributed to end-of-life costs (12 months rather than 6 as in our main analysis)

In summary, the inclusion of additional matching variables and exclusion of the 1-month pre-diagnosis period from our sepsis case definition did not substantially change our cost estimates (variations between -3% and 8%). Cost estimates were sensitive to the sepsis definitions used. Using the Sepsis-2 definition resulted in lower excess cost, 14-33% lower costs for solid tumours and 3-13% lower costs for haematology. Unsurprisingly, the proportion of end-of-life cost increased from 57% to 77% for haematology patients and from 39% to 54% for solid tumour patients as the length of terminal care increased from 6 to 12 months.

Table A11: Cumulative cost of care ($CAD 2018) results from sensitivity analysis (i)

| **Time since cancer diagnosis (months)** | **Haematology** | | | | | | | | |
| --- | --- | --- | --- | --- | --- | --- | --- | --- | --- |
|  | **Sepsis cases** | | | **Matched controls** | | | **Excess cost** | | |
|  | **Mean** | **95% CI** | | **Mean** | **95% CI** | | **Mean** | **95% CI** | |
|  |  | **LL** | **UL** |  | **LL** | **UL** |  | **LL** | **UL** |
| 1 | 19,472 | 19,163 | 19,782 | 6,603 | 6,446 | 6,761 | 12,869 | 12,528 | 13,210 |
| 3 | 35,130 | 34,751 | 35,508 | 13,485 | 13,286 | 13,685 | 21,644 | 21,221 | 22,068 |
| 6 | 54,173 | 53,713 | 54,633 | 2,568 | 2,319 | 2,817 | 1,605 | 31,094 | 32,116 |
| 12 | 79,978 | 79,391 | 80,564 | 33,759 | 33,454 | 34,064 | 46,218 | 45,577 | 46,860 |
| 24 | 109,342 | 108,662 | 110,022 | 48,391 | 48,030 | 48,752 | 60,951 | 60,193 | 61,708 |
| 60 | 158,901 | 157,970 | 159,833 | 82,589 | 82,044 | 83,134 | 76,312 | 75,239 | 77,386 |

| **Time since cancer diagnosis (months)** | **Solid tumour** | | | | | | | | |
| --- | --- | --- | --- | --- | --- | --- | --- | --- | --- |
|  | **Sepsis cases** | | | **Matched controls** | | | **Excess cost** | | |
|  | **Mean** | **95% CI** | | **Mean** | **95% CI** | | **Mean** | **95% CI** | |
|  |  | **LL** | **UL** |  | **LL** | **UL** |  | **LL** | **UL** |
| 1 | 17,194 | 16,879 | 17,510 | 9,388 | 9,223 | 9,552 | 7,807 | 7,448 | 8,166 |
| 3 | 35,001 | 34,557 | 35,445 | 21,197 | 20,960 | 21,433 | 13,804 | 13,310 | 14,298 |
| 6 | 52,024 | 51,495 | 52,553 | 32,140 | 31,851 | 32,429 | 19,884 | 19,295 | 20,473 |
| 12 | 72,206 | 71,605 | 72,808 | 43,309 | 42,977 | 43,641 | 28,898 | 28,225 | 29,570 |
| 24 | 94,157 | 93,477 | 94,838 | 53,846 | 53,482 | 54,210 | 40,311 | 39,547 | 41,076 |
| 60 | 135,036 | 134,177 | 135,896 | 73,738 | 73,270 | 74,206 | 61,298 | 60,340 | 62,257 |

Table A12: Cumulative cost of care ($CAD 2018) results from sensitivity analysis (ii)

| **Time since cancer diagnosis (months)** | **Haematology** | | | | | | | | |
| --- | --- | --- | --- | --- | --- | --- | --- | --- | --- |
|  | **Sepsis cases** | | | **Matched controls** | | | **Excess cost** | | |
|  | **Mean** | **95% CI** | | **Mean** | **95% CI** | | **Mean** | **95% CI** | |
|  |  | **LL** | **UL** |  | **LL** | **UL** |  | **LL** | **UL** |
| 1 | 20,005 | 19,683 | 20,327 | 7,064 | 6,907 | 7,220 | 12,941 | 12,581 | 13,301 |
| 3 | 36,292 | 35,883 | 36,701 | 14,442 | 14,243 | 14,641 | 21,850 | 21,387 | 22,312 |
| 6 | 56,110 | 55,614 | 56,606 | 24,067 | 23,827 | 24,307 | 32,043 | 31,493 | 32,593 |
| 12 | 82,887 | 82,285 | 83,489 | 35,557 | 35,252 | 35,861 | 47,330 | 46,654 | 48,006 |
| 24 | 112,841 | 112,143 | 113,538 | 49,950 | 49,583 | 50,317 | 62,890 | 62,104 | 63,676 |
| 60 | 164,526 | 163,611 | 165,442 | 83,061 | 82,518 | 83,605 | 81,465 | 80,389 | 82,541 |

| **Time since cancer diagnosis (months)** | **Solid tumour** | | | | | | | | |
| --- | --- | --- | --- | --- | --- | --- | --- | --- | --- |
|  | **Sepsis cases** | | | **Matched controls** | | | **Excess cost** | | |
|  | **Mean** | **95% CI** | | **Mean** | **95% CI** | | **Mean** | **95% CI** | |
|  |  | **LL** | **UL** |  | **LL** | **UL** |  | **LL** | **UL** |
| 1 | 17,819 | 17,474 | 18,164 | 9,873 | 9,699 | 10,047 | 7,946 | 7,563 | 8,330 |
| 3 | 36,262 | 35,794 | 36,730 | 22,225 | 21,974 | 22,476 | 14,037 | 13,509 | 14,565 |
| 6 | 53,806 | 53,259 | 54,353 | 33,119 | 32,823 | 33,414 | 20,688 | 20,073 | 21,302 |
| 12 | 73,758 | 73,130 | 74,386 | 43,745 | 43,406 | 44,083 | 30,013 | 29,302 | 30,725 |
| 24 | 95,619 | 94,911 | 96,326 | 53,697 | 53,318 | 54,075 | 41,922 | 41,128 | 42,716 |
| 60 | 134,361 | 133,514 | 135,208 | 72,498 | 72,032 | 72,964 | 61,863 | 60,881 | 62,844 |

Table A13: Cumulative cost of care ($CAD 2018) results from sensitivity analysis (iii)

| **Time since cancer diagnosis (months)** | **Haematology** | | | | | | | | |
| --- | --- | --- | --- | --- | --- | --- | --- | --- | --- |
|  | **Sepsis cases** | | | **Matched controls** | | | **Excess cost** | | |
|  | **Mean** | **95% CI** | | **Mean** | **95% CI** | | **Mean** | **95% CI** | |
|  |  | **LL** | **UL** |  | **LL** | **UL** |  | **LL** | **UL** |
| 1 | 17,353 | 17,025 | 17,681 | 6,530 | 6,385 | 6,676 | 10,822 | 10,458 | 11,186 |
| 3 | 32,795 | 32,386 | 33,205 | 13,289 | 13,100 | 13,479 | 19,506 | 19,050 | 19,962 |
| 6 | 51,693 | 51,196 | 52,189 | 22,127 | 21,897 | 22,358 | 29,566 | 29,009 | 30,122 |
| 12 | 76,441 | 75,834 | 77,049 | 32,507 | 32,215 | 42,798 | 43,935 | 43,244 | 44,625 |
| 24 | 103,912 | 103,215 | 104,608 | 45,373 | 45,023 | 45,722 | 58,539 | 57,736 | 59,342 |
| 60 | 153,690 | 152,784 | 154,596 | 73,602 | 73,093 | 74,110 | 80,088 | 79,038 | 81,138 |

| **Time since cancer diagnosis (months)** | **Solid tumour** | | | | | | | | |
| --- | --- | --- | --- | --- | --- | --- | --- | --- | --- |
|  | **Sepsis cases** | | | **Matched controls** | | | **Excess cost** | | |
|  | **Mean** | **95% CI** | | **Mean** | **95% CI** | | **Mean** | **95% CI** | |
|  |  | **LL** | **UL** |  | **LL** | **UL** |  | **LL** | **UL** |
| 1 | 14,284 | 14,010 | 14,559 | 9,097 | 8,932 | 9,263 | 5,187 | 4,861 | 5,513 |
| 3 | 30,789 | 30,416 | 31,163 | 20,385 | 20,142 | 20,628 | 10,404 | 9,960 | 10,848 |
| 6 | 46,505 | 46,082 | 46,929 | 30,953 | 30,664 | 31,242 | 15,553 | 15,041 | 16,064 |
| 12 | 64,746 | 64,234 | 65,257 | 41,424 | 41,088 | 41,759 | 23,322 | 22,707 | 23,937 |
| 24 | 84,259 | 83,668 | 84,851 | 50,343 | 49,969 | 50,717 | 33,916 | 33,218 | 34,614 |
| 60 | 119,377 | 118,632 | 120,121 | 66,613 | 66,171 | 67,056 | 52,763 | 51,898 | 53,628 |

Figure A4: Excess cost of care ($CAD 2018) results from sensitivity analysis (iv)

**References**

1. Levy MM, Fink MP, Marshall JC, et al. 2001 SCCM/ESICM/ACCP/ATS/SIS International Sepsis Definitions Conference. Intensive Care Med. 2003; 29: 530-8.

2. Jolley RJ, Quan H, Jetté N, et al. Validation and optimisation of an ICD-10-coded case definition for sepsis using administrative health data. BMJ open. 2015; 5: e009487.
