# Supplementary material for: Excess cost of care associated with sepsis in cancer patients: Results from a population-based case-control matched cohort
Source: PLoS One. 2021 Aug 11;16(8):e0255107. doi: 10.1371/journal.pone.0255107 (PMC8357157; doi:10.1371/journal.pone.0255107)
Supplement: Attachment — Submitted filename: PLoS One Review.docx [file pone.0255107.s010.docx]

**Title of the page**: High excess cost of care associated with sepsis in first year of cancer diagnosis: Results from a population-based case-control matched cohort article PONE-D-20-36215_reviewer.

I appreciate the authors’ curiosity to investigate economic burden of sepsis associated with cancer diagnosis and treatment. The paper shows the ice-Berge of the problem because it stands on health care perspective. But, the paper needs clarity on the following points:

1. What is the cut-off point to say **high** and **low** excess cost of care associated with sepsis?
2. When you were selecting samples, the **weight** of patient was not considered to select cases and controls, or not considering the weight of the patient was not putting as a part of study limitation. The weight of the patient could show the status of client and should be considered for case-control match sample selection. Otherwise, it is good to explain why the weight patient was not considered for case-control cross match sample selection.
3. Among Solid tumors, **others** account **42.3%** which is greater than 3% which represents large groups of solid tumor cases, to be acceptable, it is good to put others into specific solid tumors with their percentage as long as it becomes less than 3%.
4. It might not be the objective of the study, but as depicted in the table 1, the cancer diagnosis was decreased in 2017 as compared to year before 2017. Do you have any justification?
5. The discussion part of the article discusses on indirectly related parameters to the objective of the study like satisfaction of sepsis survivors, and treatment options of cancer…. It is advisable to focus on our findings of the study. E.g. the paper discusses advanced treatment options but during the study treatment modalities were not considered.

| Does the study present the results of original research? | Yes, It is the original research |
| --- | --- |
| Does the result reported have not been published elsewhere? | Yes, it is not published anywhere before |
| Does the experiments, statistics, and other analyses are performed to a high technical standard and are described in sufficient detail? | Yes, Experiments and statistical analysis were performed in high technical standard. |
| Do the conclusions are presented in an appropriate fashion and are supported by the data? | Yes, The conclusion presented in appropriate fashion. |
| Does the article is presented in an intelligible fashion and is written in Standard English? | Yes, the article is presented in an intelligible fashion and is written in Standard English. |
| Does the research meet all applicable standards for the ethics of experimentation and research integrity? | Yes, the research meets all applicable standards for the ethics of experimentation and research integrity. |
| Does the article adhere to appropriate reporting guidelines and community standards for data availability? | Yes, the article adheres to appropriate reporting guidelines and community standards for data availability. |
